# “Walking with Dreams”: The Categories of Career Decision-Making Self-Efficacy and Its Influence on Learning Engagement of Senior High School Students

**DOI:** 10.3390/bs14121174

**Published:** 2024-12-08

**Authors:** Xuejun Liu, Xiongjie Mei, Guojun Ji

**Affiliations:** 1 Institute of Education, Xiamen University, Xiamen 361005, China; 25720230156889@stu.xmu.edu.cn (X.L.); 25720210156504@stu.xmu.edu.cn (X.M.); 2 Center for Teaching and Learning Development, Xiamen University, Xiamen 361005, China; 3 Institute of Management, Xiamen University, Xiamen 361005, China

**Keywords:** career decision-making self-efficacy, learning engagement, latent profile analysis, high school students, student motivation

## Abstract

Career decision-making self-efficacy is a key factor influencing high school students’ ability to make informed choices. It is closely associated with their professional interests, learning engagement, and academic performance. This study aims to explore the latent categories of career decision-making self-efficacy among Chinese high school students and analyze the differences in learning engagement across students with different types of career decision-making self-efficacy. A convenience sampling method was employed to recruit 510 Chinese high school students as participants. A questionnaire survey was conducted using the Career Decision-Making Self-Efficacy Scale and the Learning Engagement Scale. The validity of the questionnaire was analyzed using Amos 23.0, descriptive statistics and correlation analyses were performed with SPSS 26.0, and a latent profile model was constructed using Mplus 8.0. The results indicate that there are four latent categories of career decision-making self-efficacy among high school students. In terms of learning engagement levels, the categories are ranked from highest to lowest as follows: high career decision-making self-efficacy type, lack of external exploration type, lack of internal exploration type, and low career decision-making self-efficacy type. Students with high and low career decision-making self-efficacy demonstrated significantly higher levels of learning engagement compared to those categorized as lacking external or internal exploration. Therefore, the design of career education curricula for high school students should focus on enhancing career decision-making self-efficacy to stimulate their intrinsic motivation for learning. Differences among various student types should be acknowledged, allowing for tailored and individualized instruction. Additionally, efforts should be made to strengthen integrated career guidance that links academics, career interests, and future professions, guiding all stakeholders to shift away from entrenched practices of “exam-oriented education” and utilitarian perspectives.

## 1. Introduction

In September 2014, China launched a new round of the National College Entrance Examination reforms, defined as being the largest in scale, most extensive in coverage, and most arduous in difficulty since the resumption of the National College Entrance Examination in 1977’ [1]. By 2024, the new National College Entrance Examination reform policy has been implemented for ten years, and five batches and 29 provinces and cities have launched the new National College Entrance Examination reform, and all pilot provinces and cities have focused on the reform of the subject selection system, that is, from the traditional liberal arts and science dichotomy model to the “3 + 3”, “3 + 1 + 2” election model [2]. This way expands the autonomy of colleges and universities in running schools, clarifies the dominant position of college enrollment, stimulates the vitality of ordinary high schools in running schools, and promotes the high-quality development of ordinary high schools. At the same time, this reform also increases students’ choices and brings policy support to stimulate high school students’ learning motivation, clarify the meaning of learning, and enhance the level of learning engagement [3]. However, on the one hand, due to the high-stakes nature of the National College Entrance Examination, teachers and students are trapped in utilitarian subject selection, which shows that students’ interest gives way to their scores in subject selection decisions. On the other hand, the career education and curriculum construction of Chinese high school students have not received due attention for a long time. The lack of career education makes high school students not adapt to the subject selection system of the new National College Entrance Examination reform, and there is a phenomenon that they have the right to choose but will not choose [4,5].

Career decision-making self-efficacy is the application of self-efficacy in the career field, that is, an individual’s belief in his or her ability to engage in career decision-making activities (such as collecting career information or selecting career goals) [6]. Relevant studies showed that career decision-making self-efficacy is negatively correlated with career decision-making difficulties [7,8]. In other words, the stronger the self-efficacy of senior high school students’ professional decision-making, the easier it is for them to make the optimal decision and major selection. Therefore, especially under the background of the subject selection system of the new National College Entrance Examination reform, paying attention to the self-efficacy of high school students’ professional decision-making means paying attention to the improvement of high school students’ choice ability, which is conducive to the realization of the independent choice value of the new National College Entrance Examination subject selection system.

Learning engagement is an important indicator for predicting the academic achievement of high school students and can predict the situation of students’ suspension, study, and work after ten years, which is of great significance to the long-term development of students [9]. In previous studies, the factors that affect individual learning involvement include individual characteristics, family, and school. In the study of individual characteristics, age, commitment, adaptive perfectionism, cognitive need, and cognitive motivation, learning motivation, learning interest, self-efficacy, and future orientation can significantly positively predict students’ learning engagement level [10,11,12]. In the research on the influence of family factors on learning engagement, it has been confirmed that family support, family socioeconomic status, family environment, family characteristics, parental rearing style, parental involvement in education, parental expectation, and other factors affect students’ learning engagement level [13,14]. In the research on the influence of school factors on learning engagement, it has been confirmed that teacher support, teacher-student relationship, peer communication, school atmosphere, and other factors significantly affect students’ learning engagement [15,16].

Career decision-making self-efficacy is a crucial factor in individuals’ career choice behaviors. Theories of career choice can generally be divided into two major categories. One category includes classical career choice theories, such as Parsons’ theory of person-vocation fit and Holland’s theory of vocational personalities, which emphasize the degree of congruence between individuals and their chosen careers. Career decision-making self-efficacy is a prerequisite for achieving person-vocation fit. High school students with stronger career decision-making self-efficacy are more likely to engage in self-reflection, actively explore career options, and make informed choices. The other category consists of career choice theories based on career development perspectives, represented by scholars such as Ginzberg and Super. According to Ginzberg’s career choice theory, high school students are in the tentative stage, during which adolescents begin to recognize their abilities, interests, values, and the constraints of social realities. This stage marks the gradual formation of preliminary directions for subject, major, and career choices. According to Super’s career development theory, high school students are in the exploration stage of career development, during which individuals establish career goals by exploring their vocational interests and abilities [17]. Career decision-making self-efficacy is a critical factor influencing high school students’ abilities, interests, and career exploration. Therefore, it is essential to investigate the current state and typology of career decision-making self-efficacy among high school students. Moreover, According to the social cognitive career theory, career decision-making self-efficacy can affect the interest development of high school students, thus predicting goal selection and then affecting individual activity involvement, goal achievement, and achievement performance [18]. According to Connell’s self-system theory, self-needs are significant predictors of an individual’s level of engagement [19]. The new subject selection system expands students’ autonomy, granting them greater freedom to choose subjects and enabling them to select those they are more passionate about and interested in. The new college entrance examination reform provides institutional support to meet the autonomous needs of high school students, thereby fulfilling their self-needs and promoting higher levels of learning engagement. Based on the aforementioned theoretical foundations, this study hypothesizes a relationship between career decision-making self-efficacy and learning engagement among high school students. Previous studies have shown that career decision-making self-efficacy can enhance students’ academic achievement and persistence. There is a lack of evidence-based investigation into the relationship between career decision-making self-efficacy and learning engagement among high school students. At present, China’s first new National College Entrance Examination students have been successfully enrolled into colleges and universities, but the academic research on the new National College Entrance Examination is still mainly focused on the interpretation and analysis of the comprehensive reform plan of the National College Entrance Examination, and there is a lack of empirical research related to the reform of the new National College Entrance Examination [20]. Against this background, exploring the typology of career decision-making self-efficacy among Chinese high school students and its relationship with learning engagement holds significant theoretical and practical implications.

Latent profile analysis (LPA) is a categorical latent variable modeling approach that focuses on identifying latent subpopulations within a population based on a certain set of variables [21,22]. LPA thus assumes that people can be typed with varying degrees of probabilities into categories (subpopulations) that have different configurable profiles of personal and/or environmental attributes. In particular, such categorical latent variable models allow a parsimonious representation of structures in the form of groupings [23]. Compared to traditional methods such as mean-split and cluster analysis, LPA has lower dimensionality requirements for data and relies on model fit estimation, making classification more accurate and objective [24]. LPA is an analytic strategy that has received growing interest in the work and organizational sciences in recent years [25]. A number of studies have used LPA methods to investigate issues in the work and career fields, including occupational attitudes, career success, career orientation, and professional behavior, and researchers have called for the use of LPA in the work and career fields [26,27]. The reform of the new National College Entrance Examination will advance the career decision of Chinese students to the stage of subject selection in high school, and it is necessary to cultivate students’ career and professional abilities in high school.

Therefore, under the background of the new National College Entrance Examination reform subject selection system, based on LPA, this study investigated the current situation of career decision-making self-efficacy and subgroup types of high school students and then investigated the differences in learning engagement of different subgroups. It will provide some data support and International experience for the promotion of high school students’ learning engagement level, career decision-making ability, and the improvement and development of the new National College Entrance Examination reform subject selection system.

## 2. Materials and Methods

### 2.1. Participants and Procedures

This study employed a questionnaire survey method using convenience sampling to distribute online questionnaires to high school students in H Province, China, where the new college entrance examination reform is being implemented. The questionnaire consisted of an introduction, the Career Decision-Making Self-Efficacy Scale, and the Learning Engagement Scale. Data were collected through electronic questionnaires distributed by researchers via social media platforms, including WeChat and Tencent QQ. Before participants began the survey, the instructions section informed them that the data collected would be used solely for research purposes. Participants were assured that the questionnaire was anonymous, personal privacy would not be disclosed, and the study adhered to principles of anonymity and confidentiality. Completing the questionnaire required approximately 5–8 min. The questionnaire was administered in Chinese to ensure that participants could fully understand the survey content. The study was conducted in accordance with the Declaration of Helsinki. This study involving human participants was reviewed and approved by the Ethics Committee of Xiamen University. Because there are no privacy and ethical issues involved, the Ethics Committee waived the requirement of written informed consent for participation. A total of 557 questionnaires were issued and recovered. After eliminating invalid questionnaires, 510 valid questionnaires were obtained, with an effective rate of 91.561%. The effective sample size exceeded 500, making it suitable for conducting LPA [28]. In terms of gender distribution, the valid sample consisted of 208 male students and 302 female students. Regarding grade levels, there were 185 first-year students, 112 second-year students, and 213 third-year students.

### 2.2. Measures

#### 2.2.1. Career Decision-Making Self-Efficacy Scale

This paper adopts the high school students’ Career Decision-Making Self-Efficacy Scale compiled by Peng and Long [29]. The scale consists of five dimensions, including self-evaluation, information collection, goal selection, plan-making, and problem-solving, with a total of 35 items. The five-point scoring method is adopted. The higher the score, the stronger the professional decision-making self-efficacy of high school students. In this study, the Cronbach’s alpha coefficient of the questionnaire was 0.972, and the Cronbach’s alpha coefficients of the sub-dimensions of self-evaluation, information collection, goal selection, plan making, and problem-solving were 0.900, 0.891, 0.884, 0.872 and 0.868, respectively, indicating that the scale had good reliability. The results of confirmatory factor analysis showed a good fit, with *χ*^2^/df = 1.615, *CFI* = 0.969, *TLI* = 0.967, *GFI* = 0.908, *SRMR* = 0.036, *RMSEA* = 0.035, and the scale had good validity.

#### 2.2.2. Learning Engagement Scale

Schaufel developed the Utrecht Work Engagement Scale-Student (Utrecht Work Engagement Scale-Student) based on the definition of learning engagement, which has been widely used with high validity [30]. In this study, the Chinese version of UWES-S was translated and revised by Fang to measure the learning engagement level of high school students [31]. The scale includes three dimensions of vitality, dedication, and concentration, a total of 17 questions, using a 7-point scoring method, the higher the score, the higher the level of learning engagement of high school students. In this study, Cronbach’s alpha coefficient for the questionnaire was 0.959, and Cronbach’s alpha coefficient for the vitality, dedication, and focus sub-dimensions were 0.905, 0.867, and 0.903, respectively. The scale has good reliability. The results of confirmatory factor analysis showed that the scale had good validity, with *χ*^2^/df = 1.812, *CFI* = 0.985, *TLI* = 0.982, *GFI* = 0.954, *SRMR* = 0.051, and *RMSEA* = 0.040.

### 2.3. Data Analysis

The data analysis in this study involved the following steps: First, confirmatory factor analysis (CFA) was conducted using Amos 23.0 software to assess the reliability and validity of the measurement scales. Second, SPSS 26.0 software was used to perform descriptive statistics and correlation analysis on career decision-making self-efficacy and learning engagement among high school students. Descriptive statistics provided an overview of the current levels of career decision-making self-efficacy and learning engagement, while correlation analysis revealed the relationships between the two variables and their respective factors. These results also supported the subsequent LPA. Third, Mplus 8.0 software was used to establish a latent profile model, with career decision-making self-efficacy as the manifest variable. The optimal category model was determined based on model fit indices such as the Akaike Information Criterion (AIC), Bayesian Information Criterion (BIC), and Entropy; this was used to identify the latent categories and distribution of career decision-making self-efficacy among high school students. Fourth, SPSS 26.0 software was used to analyze the differences in learning engagement among students with different categories of career decision-making self-efficacy.

In addition, prior to conducting the formal data analysis, the Harman single-factor test was used to test the common method bias, and the results showed that the explanatory variance of the first factor was 35.648%, and there was no obvious common method bias in this study [32].

## 3. Results

### 3.1. Descriptive Statistics and Correlation Analysis

As shown in Table 1, high school students have higher average scores on six factors, and their self-rated career decision-making self-efficacy and learning engagement are at a good level. Specifically, the scores of high school students’ career decision-making self-efficacy in five dimensions from high to low are in order plan making, self-evaluation, information collection, goal selection, and problem-solving. The results of correlation analysis showed that there was a moderately significant positive correlation among the six factors (0.140 ≤ r ≤ 0.867, *p* < 0.01), and there was no high correlation among the factors, which could be further cluster analysis.

### 3.2. Analysis of Potential Categories of Career Decision-Making Self-Efficacy in High School Students

#### 3.2.1. Determining the Number of Latent Profiles

Taking the five factors of professional decision self-efficacy of high school students as explicit indexes, the number of potential profile analysis categories gradually increased from one category [33]. As shown in Table 2, with the increase in the number of categories, the Loglikelihood (LL) absolute value, Akaike (AIC), Bayesian (BIC), and adjusted BIC (a BIC) of the fitting index of classification information decreased gradually from 1 to 4, that is, the model fitting gradually got better. Entropy is more than 0.90, indicating that the accuracy of these classification models is good, and the LMR and BLRT values reach significant levels. From category 4 to category 5, the values of AIC, BIC, and BIC tended to decline gently, while the values of LMR and BLRT in category 5 did not reach a significant level. Therefore, this study considers the 4 classification model as the optimal potential profile analysis model.

#### 3.2.2. Naming of Potential Profile Classes

**Figure 1:**
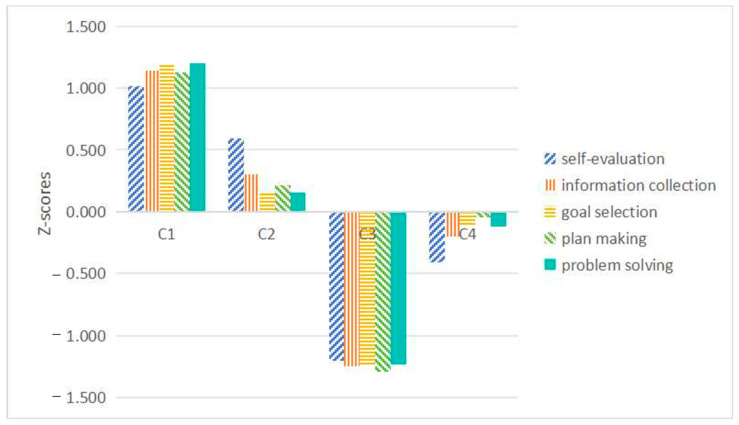
Potential profile class diagram.

Based on the scores of each category in the dimensions of self-efficacy of professional decision-making, the categories are named “high career decision-making self-efficacy type”, “lack of external exploration type”, “low career decision-making self-efficacy type”, and “lack of internal exploration type”. The proportion and standard scores of each category are shown in Table 3 and Figure 1.

The first category accounted for 60.6%, more than half of the total population. Compared with the other three types of high school students, this type of student has the highest level of career decision-making self-efficacy, has a high degree of self-assessment or confidence in the ability to complete various tasks in the course selection and major decision-making process, and can make reasonable use of the new college entrance examination subject selection system to make the most optimal subject and major decisions. High school students in this category scored highest on self-evaluation, information collection, goal selection, plan-making, and problem-solving. It shows that this kind of high school student can choose subjects and professional goals that are clear, named as high career decision-making self-efficacy type.

The second category accounted for 8.6%. This type of self-evaluation scored high, followed by information collection, plan-making, goal selection, and problem-solving. This indicates that these high school students have a clear understanding of themselves, such as their professional interests and professional abilities, but they do not explore the external environment enough. For example, the choice of majors corresponding to different subjects, the learning content of different majors, and career development are not enough, so it is difficult to make a choice, named as lack of external exploration type.

The third category accounted for 15.9%. Compared with the other three types of high school students, this type of student had the lowest scores in career decision-making self-efficacy, self-evaluation, information collection, goal selection, plan-making, and problem-solving. It shows that these high school students have unclear self-cognition, do not actively explore the external environment, and lack the ability to choose goals, make plans, and solve problems. In the face of the new entrance examination subject selection system, they are seriously uncomfortable and have difficulties in subject selection and professional decision-making, named as low career decision-making self-efficacy type.

The fourth category accounted for 14.9%. This type of self-evaluation score is lower, information collection, goal selection, plan-making, and problem-solving tend to be average. It shows that these high school students lack clear self-cognition, lack of discipline, and professional interest and need to improve their internal exploration, named as lack of internal exploration type.

Overall, the levels of career decision-making self-efficacy, ranked from highest to lowest, are as follows: high career decision-making self-efficacy type, lack of external exploration type, lack of internal exploration type, and low career decision-making self-efficacy type. Students categorized as having high career decision-making self-efficacy are characterized by the highest levels of abilities in self-assessment, information gathering, goal setting, planning, and problem-solving. Students categorized as the lack of external exploration type are characterized by relatively high self-assessment abilities but insufficient skills in information gathering, planning, goal setting, and problem-solving. Students categorized as the lack of internal exploration type are characterized by low levels of self-assessment but possess moderate abilities in information gathering, planning, goal setting, and problem-solving. Students categorized as having low career decision-making self-efficacy are characterized by the lowest levels of self-assessment, information gathering, goal setting, planning, and problem-solving abilities.

### 3.3. Different Types of High School Students in the Difference of Learning Engagement

The Kruskal-Wallis non-parametric test was used to compare the scores of learning engagement of different career decision-making self-efficacy profile groups, and the results are shown in Table 4. The levels of learning engagement from high to low were high career decision-making self-efficacy type, low career decision-making self-efficacy type, lack of external exploration type, and lack of internal exploration type. In addition, the level of learning investment of the high career decision-making self-efficacy type and low career decision-making self-efficacy type was significantly higher than that of the lack of external exploration type and lack of internal exploration type.

## 4. Conclusions and Discussion

High school students’ career decision-making self-efficacy and self-evaluation, information collection, goal selection, plan-making, and problem-solving have a significant positive correlation with their learning engagement. Internal factors are the fundamental driving force behind development. For high school students, strong learning motivation and a sense of meaning in learning are critical prerequisites for high levels of learning engagement, rooted in their intrinsic awareness and subjective initiative. The primary task of career education is to focus on the holistic development of students, emphasizing a student-centered approach. By integrating efforts from various aspects of career education, it aims to help students enhance the knowledge, skills, attitudes, and values necessary for adapting to future societal development. This enables students to achieve self-development and lifelong growth. Through career experiences, students discover their interests, clarify aspirations, establish goals, and generate motivation, thereby awakening their intrinsic strength and enhancing their level of learning engagement [34]. A review of previous studies, both domestically and internationally, indicates that internal factors such as self-efficacy, self-assessment, goal orientation, and learning strategies can significantly and positively predict high school students’ learning engagement [35,36]. Overall, career decision-making self-efficacy not only directly predicts high school students’ decision-making abilities, thereby supporting the student-centered values of the new college entrance examination, but is also associated with improvements in students’ professional interests, career choices, learning engagement, and academic performance.

Based on a typological perspective, this study employed Latent Profile Analysis (LPA) to classify high school students’ career decision-making self-efficacy. The results identified four distinct types: high career decision-making self-efficacy, lack of external exploration, lack of internal exploration, and low career decision-making self-efficacy. Previous studies have recognized career decision-making self-efficacy as an important variable and have conducted research focusing on its conceptual definition, measurement, and influencing factors. Researchers have developed career decision-making self-efficacy scales adapted to different cultural contexts [37,38]. Empirical studies have identified self-assessment, emotional intelligence, personality traits, and social support as important antecedent variables influencing individuals’ career decision-making self-efficacy [39,40,41]. Career decision-making self-efficacy can influence individuals’ career choices and processes, decision-making styles, career decision-making difficulties, and outcome expectations [42,43,44]. In addition, some scholars have employed experimental research methods to examine the effects of various career interventions on students’ career decision-making self-efficacy [45]. The above studies are conducted from a variable-centered perspective, which does not effectively capture individual heterogeneity. Due to differences in students’ personalities, family socioeconomic and cultural backgrounds, and lifestyles, disparities exist in their career awareness, knowledge, and skills, leading to variations in their professional choices and career planning for the future [46,47]. This study adopts a person-centered perspective and employs LPA to classify high school students’ career decision-making self-efficacy. This approach offers a novel perspective to enrich existing research and contributes to the development of differentiated and personalized career support for students of various types.

The results indicate that students with high and low career decision-making self-efficacy types exhibit relatively high levels of learning engagement. According to self-determination theory, intrinsic motivation refers to an individual’s engagement in an activity due to its inherent meaning and value, as well as genuine interest or enjoyment. The satisfaction of motivation originates within the activity itself rather than external factors [48]. Students with high career decision-making self-efficacy exhibit a “Walking with dreams” learning attitude, characterized by strong intrinsic motivation and high levels of learning engagement. They demonstrate a clear sense of purpose during subject selection, aligning their goals with their abilities and interests. Additionally, they possess strong problem-solving and execution skills and are more likely to engage actively in career exploration [49]. Interestingly, students with low career decision-making self-efficacy also exhibit high levels of learning engagement, a finding that appears counterintuitive and seemingly inconsistent with common expectations. The underlying reason may be that, although these students lack intrinsic motivation, they are influenced by extrinsic motivation, which drives them to exhibit relatively high levels of learning motivation and engagement. Extrinsic motivation refers to an individual’s engagement in an activity to achieve separable outcomes, such as fame, money, praise, or avoidance of punishment. The satisfaction of such motivation lies outside the activity itself rather than within it. Both intrinsic and extrinsic motivation serve to direct, stimulate, maintain, regulate, and enhance information processing, thereby contributing to the improvement of high school students’ learning engagement levels. However, the effects of intrinsic and extrinsic motivation differ. Students with strong intrinsic motivation enjoy the learning process, demonstrate persistent effort in their studies, and are better able to endure setbacks and failures. Studies have found that career decision-making self-efficacy among high school students has a significant positive impact on their career concerns and career commitment [50]. Therefore, students with high career decision-making self-efficacy and strong intrinsic motivation tend to exhibit better performance during their university years and later in their professional careers. Students with low career decision-making self-efficacy, who study primarily to achieve external goals, tend to focus on maximizing exam scores in subject selection decisions. However, once their objectives are achieved, their motivation diminishes, leading to a decline in learning engagement levels. Although these students tend to exhibit high levels of learning engagement and achieve excellent academic performance during high school, often gaining admission to prestigious universities, there is a risk that they may become “hollow individuals”. This refers to a tendency to focus solely on studying without developing other skills, resulting in insufficient motivation for development during university and potential difficulties in professional growth after entering the workforce [51].

The results indicate that students categorized as lacking external exploration and lacking internal exploration exhibit relatively low levels of learning engagement. Compared to students with high career decision-making self-efficacy, those categorized as lacking external exploration exhibit insufficient external exploration, such as a limited understanding of different universities and academic programs, indicating a need for improved environmental awareness. Students categorized as lacking internal exploration exhibit insufficient self-awareness, particularly in understanding their own interests, abilities, personality traits, and values. According to Frank Parsons’ theory of person-environment fit, individuals making career decisions need a clear understanding of their abilities and interests, as well as the requirements and conditions of different occupations [52]. Specifically, individuals first need to understand their own interests, abilities, values, and personality traits. Second, they should explore various academic disciplines and career fields to gain insight into the requirements and characteristics of different professions. Finally, they must align self-awareness with knowledge of the professional world to make appropriate academic or career choices. Students categorized as lacking external exploration or internal exploration face difficulties aligning their personal interests with professional environments, leading to challenges in subject selection decisions. These students often have unclear self-directed development goals, insufficient intrinsic learning motivation, and a lack of a sense of learning purpose, resulting in lower levels of learning engagement.

## 5. Educational Suggestion

Firstly, Focusing on enhancing career decision-making self-efficacy is key to stimulating high school students’ intrinsic learning motivation. Career decision-making self-efficacy not only directly predicts students’ decision-making abilities but is also associated with improvements in their professional interests, career choices, learning engagement, and academic performance. Therefore, the development of career education programs for high school students should focus on enhancing their career decision-making self-efficacy. Professional and systematic curriculum design is essential to improve students’ abilities across five dimensions: self-assessment, information gathering, goal setting, planning, and problem-solving. This approach aims to help students “understand themselves”, “comprehend their environment”, and “make wise choices” [53]. This approach facilitates the improvement of high school students’ career literacy, intrinsic learning motivation, and learning engagement, supporting their immediate choices and long-term development.

Secondly, Address differences among student types and implement tailored teaching approaches. By establishing a professional and authoritative career counseling platform, high school students can access services such as various assessment scales, professional counseling, and big data information. The platform can provide personalized career counseling support tailored to the unique characteristics of individual students. Schools and parents can also utilize platform data to promptly understand students’ characteristics, learning status, and psychological well-being, providing effective career guidance and support tailored to different types of students. For students with high career decision-making self-efficacy, timely tracking and support should be provided. For students lacking external exploration, relevant information on academic disciplines, institutions, and career opportunities should be promptly offered. For students lacking internal exploration, tools such as assessment scales and psychological counseling can be used to help them explore and understand themselves. For students with low career decision-making self-efficacy, guidance should focus on helping them discover their own potential and explore their environment while fostering a shift from extrinsic to intrinsic learning motivation.

Thirdly, Strengthen the integration of academic, professional, and career guidance to foster a holistic approach and guide stakeholders in shifting away from the entrenched practices of exam-oriented education and utilitarian perspectives. The subject selection reform provides students with a diversified space for choices, offering policy support to stimulate high school students’ learning motivation, clarify the significance of learning, and enhance their learning engagement. However, due to the high-stakes nature of the college entrance examination, the subject selection reform has not fully realized its intended value. Instead, it has led teachers and students into utilitarian subject selection, where decisions about subjects and career choices are primarily driven by maximizing scores rather than aligning with personal interests or life aspirations. Career planning tends to exhibit short-sighted, utilitarian, and one-dimensional tendencies, relying heavily on quantitative metrics such as income and social status to define success. Consequently, students often prioritize academic performance over personal interests in subject selection decisions [54]. In response, a top-down promotion of career education concepts is necessary to enhance career awareness and strengthen integrated guidance that encompasses academics, professional development, and career planning. This approach aims to guide students in shifting from “exam-oriented education” and “utilitarianism” to “long-term thinking” and “lifelong learning”. At the same time, efforts should be made to strengthen top-level design and improve supporting measures for career education, including legislation, curriculum development, and teacher training [55].

## 6. Limitation and Prospect

This study has certain limitations. First, due to resource constraints, the sample size of the survey was limited, and the sample lacked national representativeness. Second, the study collected cross-sectional data through self-reported questionnaires completed by high school students. This approach does not allow for the observation of changes in the latent categories of career decision-making self-efficacy over time. Regarding sample representativeness, future studies will conduct large-scale national surveys contingent on resource availability. Based on the survey results, stratified sampling will be employed to conduct in-depth interviews with students of different types to obtain more comprehensive and valuable information. In terms of research design, future studies will adopt a variable-centered perspective to incorporate new variables that influence career choices, career interests, learning engagement, and academic performance. The focus will be on exploring the mechanisms through which different types of career decision-making self-efficacy groups affect learning interests and academic achievement. Second, longitudinal studies will be conducted to reveal the transformation processes of latent categories of career decision-making self-efficacy among high school students. Additionally, follow-up investigations will examine the performance of different subgroups in subject selection, major selection, university choice, and their development during university and beyond. Finally, targeted guidance and experimental interventions will be provided for different subgroups, and the effectiveness of these interventions will be evaluated.

## Figures and Tables

**Table 1:** Descriptive Statistics and Correlation Analysis.

Variable	M	SD	1	2	3	4	5	6
1.self-evaluation	3.526	0.882	1					
2.information collection	3.522	0.870	0.867 **	1				
3.goal selection	3.520	0.881	0.832 **	0.848 **	1			
4.plan making	3.606	0.864	0.798 **	0.827 **	0.837 **	1		
5.problem solving	3.500	0.941	0.810 **	0.841 **	0.839 **	0.824 **	1	
6.learning engagement	4.768	1.203	0.145 **	0.177 **	0.142 **	0.140 **	0.159 **	1

Note: ** *p* < 0.01.

**Table 2:** Fit Indices for the Latent Profile Analysis (LPA) Model of Different Profiles.

	LL	AIC	BIC	a BIC	Entropy	pLMR	pBLRT	Class Probability
1	−3310.539	6641.078	6683.422	6651.681				1.00
2	−2111.044	4254.088	4321.839	4271.052	0.973	<0.001	<0.001	0.316/0.684
3	−1654.403	3352.805	3445.962	3376.131	0.980	<0.001	<0.001	0.212/0.163/0.625
4	−1599.014	3254.029	3372.592	3283.717	0.958	<0.001	<0.001	0.606/0.086/0.159/0.149
5	−1557.588	3183.176	3327.146	3219.225	0.965	<0.05	<0.05	0.159/0.147/0.006/0.084/0.604

Note: LL = loglikelihood; AIC = Akaike Information Criterion; BIC = Bayesian Information Criterion; a BIC = Sample-size Adjusted Bayesian Information Criterion; pLMR = *p*-value associated with the adjusted Lo–Mendel–Rubin likelihood ratio test; BLRT = Bootstrap Likelihood Ratio Test.

**Table 3:** Four types of profile tables.

	C1	C2	C3	C4
proportion	60.6%	8.6%	15.9%	14.9%
self-evaluation	1.016	0.595	−1.202	−0.409
information collection	1.144	0.305	−1.247	−0.202
goal selection	1.191	0.162	−1.242	−0.112
plan making	1.130	0.212	−1.294	−0.048
problem solving	1.200	0.154	−1.234	−0.119

Note: C1 = high career decision-making self-efficacy type, C2 = lack of external exploration type, C3 = low career decision-making self-efficacy type, C4 = lack of internal exploration type. Scores are standardized and expressed as Z-scores.

**Table 4:** Different types of learning engagement differences.

		C1	C2	C3	C4
learning engagement	mean	291.581	175.091	266.469	143.664
	H	75.902			
	*p*	<0.001			
	multiple comparisons	C1 > C2, C4; C3 > C2, C4			

Note: C1 = high career decision-making self-efficacy type, C2 = lack of external exploration type, C3 = low career decision-making self-efficacy type, C4 = lack of internal exploration type.

## Data Availability

The original contributions presented in the study are included in the Supplementary Materials, further inquiries can be directed to the corresponding author. Further inquiries can be directed to the corresponding author.

## Supplementary Materials

The following supporting information can be downloaded at: https://www.mdpi.com/article/10.3390/bs14121174/s1.
