# Correction: Liu et al. (2024). “Walking with Dreams”: The Categories of Career Decision-Making Self-Efficacy and Its Influence on Learning Engagement of Senior High School Students. *Behavioral Sciences*, *14*(12), 1174

**DOI:** 10.3390/bs15010011

**Published:** 2024-12-27

**Authors:** Xuejun Liu, Xiongjie Mei, Guojun Ji

**Affiliations:** 1 Institute of Education, Xiamen University, Xiamen 361005, China; 25720230156889@stu.xmu.edu.cn (X.L.); 25720210156504@stu.xmu.edu.cn (X.M.); 2 Center for Teaching and Learning Development, Xiamen University, Xiamen 361005, China; 3 Institute of Management, Xiamen University, Xiamen 361005, China

The authors would like to adjust the author order for the following two reasons: 1. Xiongjie Mei was the second author in the original submission and 2. Xiongjie Mei provided constructive comments that significantly enhanced the quality and depth of the paper. The authors state that the scientific conclusions are unaffected. This correction was approved by the Academic Editor. The original publication (Liu et al., 2024) has also been updated.

## References

[B1-behavsci-15-00011] Liu X., Mei X., Ji G. (2024). “Walking with Dreams”: The Categories of Career Decision-Making Self-Efficacy and Its Influence on Learning Engagement of Senior High School Students. Behavioral Sciences.

